# Effects of External Radiation Exposure Resulting From the Fukushima Daiichi Nuclear Power Plant Accident on the Health of Residents in the Evacuation Zones: the Fukushima Health Management Survey

**DOI:** 10.2188/jea.JE20210286

**Published:** 2022-12-05

**Authors:** Akira Sakai, Masanori Nagao, Hironori Nakano, Tetsuya Ohira, Tetsuo Ishikawa, Mitsuaki Hosoya, Michio Shimabukuro, Atsushi Takahashi, Junichiro J. Kazama, Kanako Okazaki, Fumikazu Hayashi, Seiji Yasumura, Hitoshi Ohto, Kenji Kamiya

**Affiliations:** 1 Department of Radiation Life Sciences, Fukushima Medical University School of Medicine, Fukushima, Japan; 2 Department of Epidemiology, Fukushima Medical University School of Medicine, Fukushima, Japan; 3 Department of Radiation Physics and Chemistry, Fukushima Medical University School of Medicine, Fukushima, Japan; 4 Department of Pediatrics, Fukushima Medical University School of Medicine, Fukushima, Japan; 5 Department of Diabetes, Endocrinology and Metabolism, Fukushima Medical University School of Medicine, Fukushima, Japan; 6 Department of Gastroenterology, Fukushima Medical University School of Medicine, Fukushima, Japan; 7 Department of Nephrology and Hypertension, Fukushima Medical University School of Medicine, Fukushima, Japan; 8 Department of Physical Therapy, Fukushima Medical University School of Health Sciences, Fukushima, Japan; 9 Department of Public Health, Fukushima Medical University School of Medicine, Fukushima, Japan; 10 Radiation Medical Science Center for the Fukushima Health Management Survey, Fukushima Medical University, Fukushima, Japan

**Keywords:** comprehensive health check, external radiation exposure dose, lifestyle-related disease, lymphopenia, neutropenia

## Abstract

**Background:**

Associations have been reported between lifestyle-related diseases and evacuation after the Great East Japan Earthquake (GEJE). However, the relationship between lifestyle-related diseases and the effective radiation dose due to external exposure (EDEE) after the GEJE remains unclear.

**Methods:**

From among 72,869 residents of Fukushima Prefecture (31,982 men; 40,887 women) who underwent a comprehensive health check in fiscal year (FY) 2011, the data of 54,087 residents (22,599 men; 31,488 women) aged 16 to 84 years were analyzed. The EDEE data of 25,685 residents with incomplete results from the basic survey, performed to estimate the external radiation exposure dose, were supplemented using multiple imputation. The data were classified into three groups based on EDEE (0 to <1, 1 to <2, and ≥2 mSv groups and associations between the incidence of diseases and EDEE from FY2011 to FY2017 were examined using a Cox proportional hazards model, with FY2011 as the baseline.

**Results:**

A higher EDEE was associated with a greater incidence of hypertension, diabetes mellitus, dyslipidemia, hyperuricemia, liver dysfunction, and polycythemia from FY2011 to FY2017 in the age- and sex-adjusted model. However, after further adjustment for evacuation status and lifestyle-related factors, the significant associations disappeared. No association was found between EDEE and other lifestyle-related diseases.

**Conclusion:**

EDEE was not directly associated with the incidence of lifestyle-related diseases after the GEJE. However, residents with higher external radiation doses in Fukushima Prefecture might suffer from lifestyle-related diseases related to evacuation and the resultant lifestyle changes.

## INTRODUCTION

To investigate the effects of radiation exposure resulting from the 2011 Great East Japan Earthquake (GEJE) and the subsequent accidents at the Fukushima Daiichi Nuclear Power Plant (FDNPP), the government of Fukushima Prefecture conducted external radiation exposure dose estimations based on movement surveys, referred to as the basic survey, for all residents of the prefecture covering a 4-month period after the GEJE (March 11, 2011 to July 11, 2011).^1^^,^^2^ As of March 31, 2019, the response rate was approximately 28%.^3^ An analysis of 421,394 individuals, excluding radiation workers and residents with movement records covering less than 4 months, from among 26.4% (541,653 of 2,055,533) of the target residents of Fukushima Prefecture as of June 30, 2014, revealed that 99.4% of the residents had an effective radiation dose due to external exposure (EDEE) of <3 mSv for the first 4 months after the GEJE, with mean and maximum exposures of 0.8 mSv and 25 mSv, respectively.^3^^,^^4^

We previously examined the results of a comprehensive health check (CHC), which is part of the Fukushima Health Management Survey, of residents of 13 municipalities who were forced by the government to evacuate due to the GEJE and subsequent accidents at the FDNPP.^1^^,^^5^ Our findings demonstrated that evacuation was a risk factor for obesity,^6^ hypertension,^7^^,^^8^ diabetes mellitus (DM),^9^^,^^10^ hypo-high-density lipoprotein cholesterolemia,^11^ metabolic syndrome,^12^ kidney disease,^13^ liver dysfunction,^14^ polycythemia,^15^^,^^16^ and hyperuricemia.^17^^,^^18^ Furthermore, no differences were found in white blood cell (WBC) counts (neutrophil and lymphocyte counts) within 1 year after the GEJE according to the evacuation area.^19^

Although only about one-fourth of the target residents have been analyzed, we speculated that the estimated EDEE is not expected to be of concern in terms of its health effects, because the EDEE of ≥90% in the residents was <3 mSv,^3^^,^^4^ which is the same level of the average individual ionizing radiation dose per year from all natural radiation sources, approximately 2.4 mSv.^20^ However, to the best of our knowledge, the relationships between individual EDEEs and the results of the CHC have not been investigated. This study aimed to investigate the relationship between them in residents of Fukushima Prefecture. For residents with incomplete results from the movement survey for 4 months after the GEJE, we supplemented the residents’ EDEE data with similar evacuation movements using multiple imputation (MI).

## METHODS

### Study population and design

**Figure 1.**
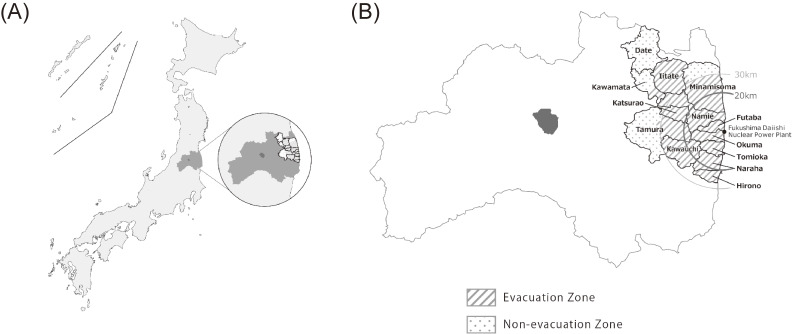
Locations of the government-designated evacuation zones. (A) Location of Fukushima prefecture in Japan. (B) Locations of the 13 municipalities in the evacuation zones in Fukushima prefecture. Areas that were completely evacuated after the Great East Japan Earthquake (GEJE) are indicated by gray lines.

**Figure 2.**
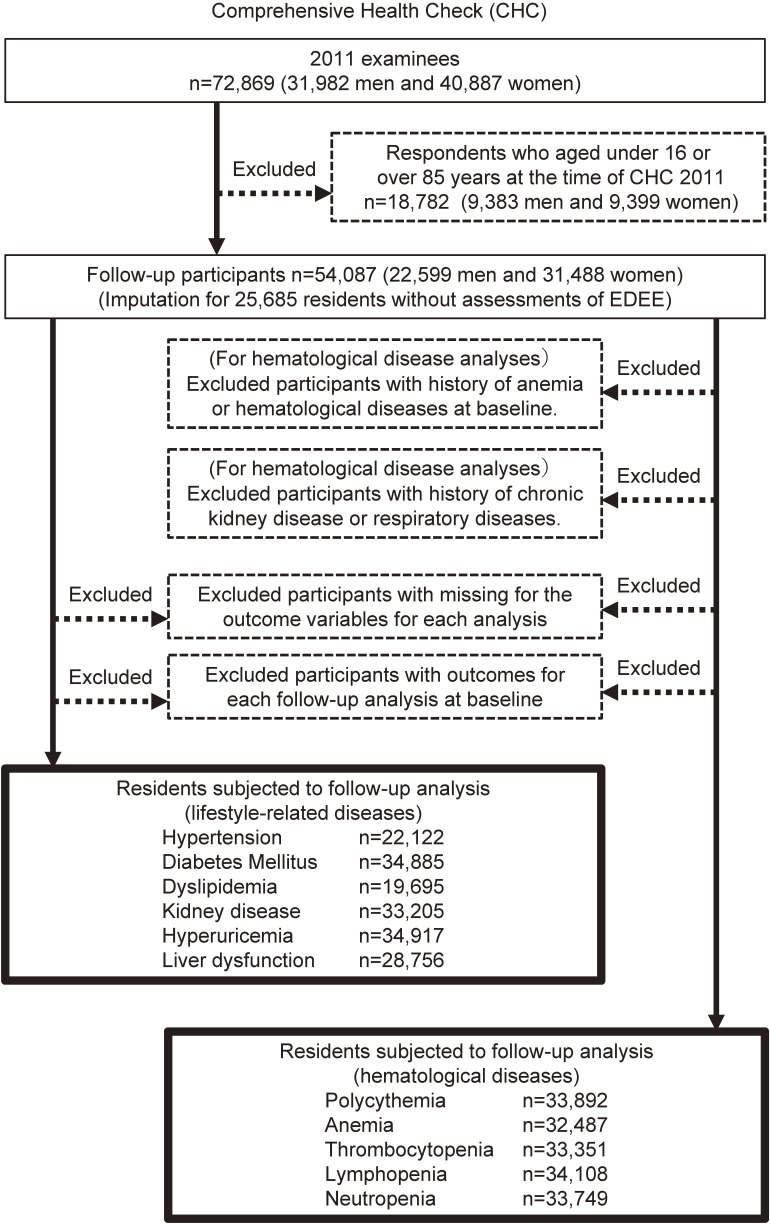
Flow diagram displaying how individuals were recruited and studied. Detailed descriptions are provided in the text.

The subjects of this study were almost 210,000 residents of all ages living in the following communities near the FDNPP in Fukushima prefecture: Tamura, Minami-Soma, Kawamata, Hirono, Naraha, Tomioka, Kawauchi, Okuma, Futaba, Namie, Katsurao, Iitate, and Date (Figure 1). All the residents of Hirono, Naraha, Tomioka, Kawauchi, Okuma, Futaba, Namie, Katsurao, and Iitate, and some of the residents of Tamura, Minami-Soma, Kawamata, and Date, were forced to evacuate their homes after the disaster due to a government order. The subjects thereafter received (1) annual health check-ups with additional items in specified health check-ups conducted by each municipality, (2) annual group health check-ups conducted by Fukushima Medical University or (3) individual health check-ups at designated medical institutions in and outside Fukushima Prefecture. Detailed methods of the CHC have been reported elsewhere.^1^^–^^3^^,^^21^ The number of medical examinees in fiscal year (FY) 2011 was 72,869 (31,982 men; 40,887 women), and the participation rate for the initial census population was 30.9% for people ≥16 years old. We excluded 18,782 examinees who were <16 or ≥85 years of age as of March 11, 2011 from the study. Therefore, 54,087 (22,599 men; 31,488 women; Figure 2) were included in the baseline analysis.

Follow-up medical examinations were conducted annually from FY2012 through FY2017 as part of the CHC. Since the evacuees had moved all over the country, the follow-up medical examination was conducted by the designated medical institutions nationwide if the evacuees were outside the prefecture. We excluded participants who had missing outcome variables for each analysis, or outcomes for each analysis at FY2011 CHC as baseline. For hematological diseases, we excluded participants with a history of anemia or hematological disorders at baseline, and chronic renal failure or respiratory diseases at each CHC. The number of participants who received follow-up examinations for each of the outcomes evaluated is shown in Figure 2. Approval was obtained from community representatives for performance of this epidemiologic study according to the guidelines of the Council for International Organizations of Medical Science, and the study was also approved by the Ethics Committee of Fukushima Medical University (#1319).

### Estimation of EDEE

The basic survey was conducted to estimate EDEE in people who were registered residents of Fukushima Prefecture from March 11 to July 1, 2011. Details of the basic survey are described elsewhere.^4^ Briefly, it was a self-administered questionnaire survey that asked the residents to record and send back information on their behavior (including time spent indoors and outdoors and time of moves) in the first 4 months after the accident (March 11, 2011). The respondents’ behavior records were digitized, and a computer program calculated individual EDEEs by superimposing the behavior records with daily ambient dose rate maps. The dose rate maps were based on available measurement data and created as a 2 km^2^ mesh. The dose rate was assumed to be uniform within each mesh area. Considering the above method, the calculation of EDEEs is considered to have some amount of uncertainty related to the dose rate maps, as well as the residents’ memory about their behavior. Since the basic survey was considered the only method for estimating individual external doses in the early stage after the accident, it is difficult to check the accuracy of EDEE by comparing it with that estimated using another method. However, municipality-wide average doses estimated from the basic survey results were in reasonable agreement with personal dosimeter measurements, as described in another paper in the same supplement issue.^3^ The overall response rate to the questionnaire to estimate external doses was approximately 28%, although the rate was different among municipalities.^3^ We also confirmed that the estimated external doses were representative of the dose distribution for the entire population of each municipality.^22^

### Multiple imputation for EDEE

Dose estimation was performed based on body size (child or adult) and the daily gamma ray dose rate at the point where the subject stayed during the 4 months after the GEJE. The atmospheric radiation dose was the highest for 2 weeks after the GEJE, and evacuation was often carried out during this period. Therefore, information on whether or not the residents evacuated and their evacuation route was important for estimation of EDEE. Evacuation routes and timings for residents in the areas forced to evacuate can be divided into several scenarios^23^ that are associated with the area of residence at the time of the GEJE and the evacuation site. Since 25,685 of the subjects (47.5%) did not participate in the basic survey performed to estimate the external radiation exposure dose in the subjects of the CHC, we used MI by chained equations with predictive mean matching methods under fully conditional specifications to generate 10 datasets for imputation of missing data on the EDEE.^24^^–^^26^ Finally, the following variables were used as covariates in the imputation model, in addition to EDEE: sex, age at the time of the disaster, and area of residence (at the time of the GEJE and for half a year after the GEJE).

### Measurements and definitions

Systolic and diastolic blood pressure were measured in accordance with previous studies.^7^^,^^8^ Hypertension was defined as systolic blood pressure ≥140 mm Hg, diastolic blood pressure ≥90 mm Hg, or the use of antihypertensive medication. The glycated hemoglobin (HbA1c) level of each patient was estimated according to National Glycohemoglobin Standardization Program guidelines, and the equivalent value was calculated using the following formula:

$$\begin{array}{rl} \text{HbA1c (\%)} & =1.019\times \text{HbA1c (Japanese Diabetes Society) (\%)}\\ & \;+0.30\%.^{27} \end{array}$$

According to the Japanese Diabetes Society Committee diagnostic criteria for diabetes,^27^ DM was defined as a fasting plasma glucose level ≥126 mg/dL (7.0 mmol/L), casual blood plasma glucose level ≥200 mg/dL, HbA1c level ≥6.5%, or the self-reported use of antihyperglycemic agents. Subjects with an HbA1c of 6.5% or higher were considered to have DM even if their fasting blood glucose was less than 126 mg/dL. Liver dysfunction was defined as an aspartate aminotransferase or alanine aminotransferase level ≥31 IU/L, or a gamma-glutamyl transpeptidase level ≥51 IU/L.^28^ Dyslipidemia was defined as high-density lipoprotein cholesterol level <40 mg/dL or low-density lipoprotein cholesterol ≥140 mg/dL in accordance with Japan Atherosclerosis Society guidelines.^29^ Serum creatinine was assayed using an enzymatic method. Estimated glomerular filtration rate (eGFR) was calculated using the following formula recommended by the Japanese Society of Nephrology^30^:

$$\begin{array}{rl} \text{eGFR} & =194\times {\text{serum creatinine}}^{-1.094}\\ & \;\times {\text{age}}^{-0.287}\;(\times \text{ 0.739 for women}). \end{array}$$

In the present study, chronic kidney disease was defined as an eGFR <60 mL/min/1.73 m^2^. Since there are different cutoff values for hyperuricemia depending on sex, hyperuricemia was defined as a serum uric acid level >7.0 mg/dL for men and >6.0 mg/dL for women.^18^ The quantitative definition of polycythemia differs between research institutes and laboratories. In the CHC, the standard values for peripheral blood were as follows: in men, red blood cell (RBC) count of 400–579 × 10^4^/µL, hemoglobin (Hb) level of 13.1–17.9 g/dL, and hematocrit (Ht) of 38.0–54.9%; and in women, RBC of 370–549 × 10^4^/µL, Hb level of 12.1–15.9 g/dL, and Ht of 33.0–47.9%. Polycythemia was diagnosed if any one of these items exceeded the standard value. In the CHC, anemia was defined as an Hb level of ≤13.1 g/dL in men and ≤12.1 g/dL in women. Thrombocytopenia was defined as platelet count (PLT) <15 × 10^4^/µL. Lymphopenia and neutropenia were defined as lymphocyte count <1,000/µL and neutrophil count <1,500/µL, respectively. Height in stockinged feet and weight in light clothing were measured. Body mass index (BMI) was calculated as weight (kg)/height (m)^2^, and obesity was defined as BMI ≥25 kg/m^2^. We obtained histories of cigarette smoking and weekly alcohol intake in “*go*” units, which is a traditional Japanese unit of volume corresponding to 20 g of ethanol, which was subsequently converted to g/day. Participants who consumed ≥40 g of ethanol per day were classified as heavy drinkers.

### Statistical analysis

Subjects’ baseline characteristics are shown as the average of the 10 imputed datasets (Table 1, Table 2, eTable 1). For the follow-up survey, Cox proportional hazards model analyses were conducted individually on all imputed data sets, and the results were combined using Rubin’s rule^20^ to obtain the overall hazard ratios (HRs) and 95% confidence intervals (CIs) of the effect of the estimated EDEE on the incidence of the various outcomes from FY2012 to FY2017, with FY2011 as the baseline. The subjects’ data were classified according to EDEE into 0 to <1, 1 to <2, and ≥2 mSv groups. The covariates in the Cox proportional hazards model analysis were the following parameters at baseline: age (continuous), sex, smoking status (current smoker), heavy drinking (alcohol equivalent ≥40 g per day), evacuation status, and hemodynamic parameters and blood test value(s) (ie, hypertension: systolic blood pressure <120, 120–129, 130–139 and diastolic blood pressure <80, 80–84, 85–90, DM: HbA1c, dyslipidemia: HDL cholesterol and LDL cholesterol, kidney disease: eGFR, polycythemia: RBC, Hb and Ht, anemia: Hb, thrombocytopenia: platelet count, lymphopenia: lymphocyte count, and neutropenia: neutrophil count). The values of hemodynamic and biochemical parameters excluding blood pressure were divided into quartiles for the subjects participating in each analyses, while evacuation status was defined based on living in the evacuation area (all areas of Hirono, Naraha, Tomioka, Kawauchi, Okuma, Futaba, Namie, Katsurao and Iitate, and part of Tamura, Minami-Soma, Kawamata, and Date), and municipalities where evacuation orders were lifted in most areas of the municipality by the end of 2016 (Hirono, Date, Tamura, Naraha, Kawauchi, Katsurao, and Minami-Soma) at the time of the disaster. BMI (quartiles) and medication (for hypertension, DM, or dyslipidemia) were also adjusted for lifestyle-related diseases (hypertension, DM, dyslipidemia, kidney disease, hyperuricemia, and liver dysfunction). Since lifestyle-related diseases are greatly affected by BMI and evacuation, mediation analysis was performed for analyses of lifestyle-related diseases using five adjusted models, which incorporated the following covariates: model 1, age and sex; model 2a, model 1 and BMI; model 2b, model 1 and evacuation status; model 3, model 1, BMI, and evacuation status; model 4, all of covariates in the main analyses (eTable 2). These analyses were also performed among the participants with complete data in the basic survey for sensitivity analyses.

**Table 1.** Baseline characteristics of the study subjects according to assessment status of EDEE

	ALL	No assessment	Assessed
Subjects, *n* (%)	54,087 (100.0)	25,685 (47.5)	28,402 (52.5)
Men, *n* (%)	22,599 (41.8)	10,796 (42.0)	11,803 (41.6)
Age at baseline, years, mean (SD)	52.5 (18.2)	51.7 (18.5)	53.2 (17.8)
BMI, kg/m^2^, mean (SD)	23.6 (3.8)	23.6 (3.8)	23.5 (3.7)
Systolic BP, mm Hg, mean (SD)	126.5 (17.5)	126.6 (17.7)	126.5 (17.4)
Diastolic BP, mm Hg, mean (SD)	76.1 (11.4)	76.2 (11.5)	76.1 (11.2)
HDL cholesterol, mg/dL, mean (SD)	60.5 (15.2)	60.1 (15.2)	60.8 (15.2)
LDL cholesterol, mg/dL, mean (SD)	122.4 (33.0)	122.4 (33.3)	122.3 (32.7)
TG, mg/dL, mean (SD)	109.1 (81.9)	109.3 (82.7)	108.9 (81.2)
HbA1c, %, mean (SD)	5.4 (0.7)	5.4 (0.8)	5.4 (0.7)
Uric acid, mg/dL, mean (SD)	5.0 (1.4)	5.0 (1.4)	5.0 (1.4)
eGFR, mL/min/1.73 m^2^, mean (SD)	78.8 (17.4)	79.1 (17.4)	78.5 (17.4)
AST, IU/L, mean (SD)	23.7 (13.4)	23.6 (13.6)	23.8 (13.3)
ALT, IU/L, mean (SD)	22.8 (21.7)	22.8 (23.0)	22.8 (20.5)
γ-GTP, IU/L, mean (SD)	33.9 (47.5)	34.1 (50.1)	33.8 (45.0)
RBC, ×10^4^/µL, mean (SD)	470 (45)	471 (45)	469 (45)
Hb, g/dL, mean (SD)	14.2 (1.6)	14.3 (1.6)	14.2 (1.6)
Ht, %, mean (SD)	42.7 (4.1)	42.7 (4.1)	42.7 (4.0)
WBC, ×10^3^/µL, mean (SD)	6.0 (1.7)	6.0 (1.7)	5.9 (1.6)
PLT, ×10^4^/µL, mean (SD)	25.0 (6.1)	25.2 (6.2)	24.8 (6.0)
Lymphocyte, count/µL, mean (SD)	2,129 (662)	2,147 (657)	2,112 (666)
Neutrophil, count/µL, mean (SD)	3,314 (1282)	3,345 (1295)	3,285 (1269)
Obesity, *n* (%)	17,132 (31.7)	8,282 (32.2)	8,850 (31.2)
Hypertension, *n* (%)	21,220 (39.2)	9,944 (38.7)	11,276 (39.7)
DM, *n* (%)	4,916 (9.1)	2,352 (9.2)	2,564 (9.0)
Dyslipdemia, *n* (%)	5,356 (9.9)	2,245 (8.7)	3,111 (11.0)
Kidney disease, *n* (%)	6,919 (12.8)	3,274 (12.7)	3,645 (12.8)
Hyperuricemia, *n* (%)	6,513 (12.0)	3,127 (12.2)	3,386 (11.9)
Liver dysfunction, *n* (%)	14,562 (26.9)	6,870 (26.7)	7,692 (27.1)
Polycythemia, *n* (%)	964 (1.8)	498 (1.9)	466 (1.6)
Anemia, *n* (%)	2,925 (5.4)	1,383 (5.4)	1,542 (5.4)
Thrombocytopenia, *n* (%)	1,126 (2.1)	506 (2.0)	620 (2.2)
Lymphopenia, *n* (%)	578 (1.1)	246 (1.0)	332 (1.2)
Neutropenia, *n* (%)	1,001 (1.9)	443 (1.7)	558 (2.0)
Smoking habitus, *n* (%)	10,096 (18.7)	5,391 (21.0)	4,705 (16.6)
Heavy drinking, *n* (%)	2,672 (4.9)	1,309 (5.1)	1,363 (4.8)
Living in the following areas at the time of disaster			
Evacuation areas,^a^ *n* (%)	27,080 (50.1)	10,456 (40.7)	16,624 (58.5)
Evacuation order lifted by the end of 2016,^b^ *n* (%)	32,738 (60.5)	16,387 (63.8)	16,351 (57.6)
Medication			
Hypertension, *n* (%)	14,242 (26.3)	6,533 (25.4)	7,709 (27.1)
DM, *n* (%)	2,778 (5.1)	1,335 (5.2)	1,443 (5.1)
Dyslipidemia, *n* (%)	7,139 (13.2)	3,072 (12.0)	4,067 (14.3)

BMI, body mass index; BP, blood pressure; EDEE, effective radiation dose due to external exposure; HDL, high-density lipoprotein; LDL, low-density lipoprotein; TG, triglyceride; FBG, fasting blood glucose; eGFR, estimated glomerular filtration rate; AST, aspartate aminotransferase; ALT, alanine aminotransferase; γ-GTP, gamma-glutamyl transpeptidase; RBC, red blood cells; Hb, hemoglobin; Ht, hematocrit; WBC, white blood cells; PLT, platelets; DM, diabetes mellitus; SD, standard deviation.

The numbers of people represent the average of the 10 imputed datasets.

^a^All area of Hirono, Naraha, Tomioka, Kawauchi, Okuma, Futaba, Namie, Katsurao, and Iitate, and part of Tamura, Minami-Soma, Kawamata, and Date.

^b^Hirono, Date, Tamura, Naraha, Kawauchi, Katsurao and Minami-Soma.

**Table 2.** Baseline characteristics of the study subjects according to EDEE assessment status subdivided into the three EDEE groups

Effective dose	No assessment (imputed EDEE) (*n* = 25,685)			Assessed (*n* = 28,402)		
	
	<1 mSv	1 to <2 mSv	≥2 mSv	<1 mSv	1 to <2 mSv	≥2 mSv
Subjects, *n* (%)	17,287 (67.3)	6,226 (24.2)	2,173 (8.5)	19,238 (67.7)	7,089 (25.0)	2,075 (7.3)
Men, *n* (%)	6,657 (38.5)	3,041 (48.8)	1,098 (50.5)	7,568 (39.3)	3,123 (44.1)	1,112 (53.6)
Age at baseline, years, mean (SD)	51.6 (18.5)	51.6 (18.5)	52.2 (18.0)	52.8 (18.0)	53.7 (17.7)	55.2 (16.2)
BMI, kg/m^2^, mean (SD)	23.6 (3.8)	23.7 (3.8)	24.0 (3.9)	23.4 (3.7)	23.6 (3.7)	24.4 (3.8)
Systolic BP, mm Hg, mean (SD)	126.3 (17.7)	127.0 (17.8)	127.5 (17.8)	125.8 (17.3)	127.5 (17.5)	129.2 (16.8)
Diastolic BP, mm Hg, mean (SD)	75.9 (11.5)	76.6 (11.5)	76.9 (11.6)	75.6 (11.3)	76.9 (11.1)	78.5 (11.1)
HDL cholesterol, mg/dL, mean (SD)	60.4 (15.1)	59.9 (15.2)	59.3 (15.2)	61.0 (15.2)	60.8 (15.5)	58.6 (15.0)
LDL cholesterol, mg/dL, mean (SD)	122.0 (33.1)	123.1 (33.8)	123.1 (34.2)	121.7 (32.7)	123.6 (32.6)	123.9 (32.1)
TG, mg/dL, mean (SD)	107.6 (80.0)	111.8 (88.8)	116.1 (84.8)	107.4 (78.9)	108.7 (77.7)	124.2 (108.6)
HbA1c, %, mean (SD)	5.4 (0.7)	5.4 (0.8)	5.4 (0.8)	5.4 (0.7)	5.4 (0.7)	5.5 (0.8)
Uric acid, mg/dL, mean (SD)	4.9 (1.4)	5.1 (1.4)	5.1 (1.4)	5.0 (1.4)	5.0 (1.4)	5.2 (1.4)
eGFR, mL/min/1.73 m^2^, mean (SD)	78.8 (17.4)	79.3 (17.4)	80.4 (17.2)	78.7 (17.6)	78.1 (17.0)	78.4 (16.7)
AST, IU/L, mean (SD)	23.4 (13.5)	23.9 (13.9)	24.9 (13.7)	23.6 (13.1)	23.7 (11.2)	25.6 (19.6)
ALT, IU/L, mean (SD)	22.3 (23.1)	23.4 (23.1)	24.8 (21.5)	22.5 (20.6)	22.7 (18.9)	26.3 (23.9)
γ-GTP, IU/L, mean (SD)	32.9 (46.8)	36.1 (52.4)	38.6 (65.9)	32.9 (44.0)	34.2 (45.2)	40.6 (52.8)
RBC, ×10^4^/µL, mean (SD)	469 (45)	476 (46)	476 (46)	468 (45)	472 (44)	476 (46)
Hb, g/dL, mean (SD)	14.2 (1.6)	14.5 (1.6)	14.5 (1.6)	14.1 (1.6)	14.3 (1.6)	14.6 (1.6)
Ht, %, mean (SD)	42.5 (4.1)	43.2 (4.1)	43.3 (4.1)	42.5 (4.0)	42.8 (4.0)	43.5 (4.0)
WBC, ×10^3^/µL, mean (SD)	6.0 (1.7)	6.1 (1.7)	6.1 (1.7)	5.9 (1.6)	6.0 (1.7)	6.1 (1.7)
PLT, ×10^4^/µL, mean (SD)	25.3 (6.3)	25.1 (6.1)	25.0 (6.0)	24.8 (5.9)	25.0 (6.2)	24.8 (6.1)
Lymphocyte, count/µL, mean (SD)	2,140 (655)	2,161 (657)	2,163 (674)	2,105 (640)	2,119 (736)	2,149 (657)
Neutrophil, count/µL, mean (SD)	3,321 (1,286)	3,397 (1,316)	3,392 (1,305)	3,265 (1,262)	3,316 (1,271)	3,367 (1,322)
Obesity, *n* (%)	5,438 (31.5)	2,058 (33.1)	787 (36.2)	5,697 (29.6)	2,277 (32.1)	876 (42.2)
Hypertension, *n* (%)	6,593 (38.1)	2,462 (39.6)	889 (40.9)	7,332 (38.1)	2,973 (41.9)	971 (46.8)
DM, *n* (%)	1,539 (8.9)	606 (9.7)	206 (9.5)	1,669 (8.7)	678 (9.6)	217 (10.5)
Dyslipdemia, *n* (%)	1,553 (9.0)	522 (8.4)	171 (7.9)	2,162 (11.2)	755 (10.7)	194 (9.3)
Kidney disease, *n* (%)	2,257 (13.1)	775 (12.4)	242 (11.2)	2,543 (13.2)	875 (12.3)	227 (10.9)
Hyperuricemia, *n* (%)	2,019 (11.7)	817 (13.1)	291 (13.4)	2,218 (11.5)	869 (12.3)	299 (14.4)
Liver dysfunction, *n* (%)	4,384 (25.4)	1,786 (28.7)	701 (32.3)	4,955 (25.8)	1,973 (27.8)	764 (36.8)
Polycythemia, *n* (%)	308 (1.8)	142 (2.3)	49 (2.2)	320 (1.7)	101 (1.4)	45 (2.2)
Anemia, *n* (%)	974 (5.6)	295 (4.7)	114 (5.2)	1,087 (5.7)	358 (5.1)	97 (4.7)
Thrombocytopenia, *n* (%)	322 (1.9)	123 (2.0)	61 (2.8)	424 (2.2)	151 (2.1)	45 (2.2)
Lymphopenia, *n* (%)	162 (0.9)	62 (1.0)	22 (1.0)	229 (1.2)	81 (1.1)	22 (1.1)
Neutropenia, *n* (%)	309 (1.8)	95 (1.5)	39 (1.8)	390 (2.0)	128 (1.8)	40 (1.9)
Smoking habitus, *n* (%)	3,435 (19.9)	1,430 (23.0)	526 (24.2)	3,061 (15.9)	1,179 (16.6)	465 (22.4)
Heavy drinking, *n* (%)	798 (4.6)	368 (5.9)	143 (6.6)	854 (4.4)	345 (4.9)	164 (7.9)
Living in the following areas at the time of disaster						
Evacuation areas,^a^ *n* (%)	6,244 (36.1)	2,617 (42.0)	1,596 (73.4)	11,116 (57.8)	3,769 (53.2)	1,739 (83.8)
Evacuation order lifted by the end of 2016,^b^ *n* (%)	12,355 (71.5)	3,363 (54.0)	668 (30.8)	12,373 (64.3)	3,438 (48.5)	540 (26.0)
Medication						
Hypertension, *n* (%)	4,378 (25.3)	1,586 (25.5)	568 (26.2)	5,050 (26.3)	2,022 (28.5)	637 (30.7)
DM, *n* (%)	883 (5.1)	348 (5.6)	105 (4.8)	964 (5.0)	372 (5.2)	107 (5.2)
Dyslipidemia, *n* (%)	2,075 (12.0)	743 (11.9)	254 (11.7)	2,814 (14.6)	989 (14.0)	264 (12.7)

BMI, body mass index; BP, blood pressure; EDEE, effective radiation dose due to external exposure; HDL, high-density lipoprotein; LDL, low-density lipoprotein; TG, triglyceride; FBG, fasting blood glucose; eGFR, estimated glomerular filtration rate; AST, aspartate aminotransferase; ALT, alanine aminotransferase; γ-GTP, gamma-glutamyl transpeptidase; RBC, red blood cells; Hb, hemoglobin; Ht, hematocrit; WBC, white blood cells; PLT, platelets; DM, diabetes mellitus; SD, standard deviation.

The numbers of people represent the average of the 10 imputed datasets.

^a^All area of Hirono, Naraha, Tomioka, Kawauchi, Okuma, Futaba, Namie, Katsurao, and Iitate, and part of Tamura, Minami-Soma, Kawamata, and Date.

^b^Hirono, Date, Tamura, Naraha, Kawauchi, Katsurao and Minami-Soma.

Statistical analyses were conducted using SAS version 9.4 (SAS Institute, Cary, NC, USA), the MI procedure was used to create multiple imputed data sets, and the MI analysis procedure was used to combine the results of the analyses of imputations. All tests were two-tailed, and *P*-values <0.05 were considered to indicate statistical significance.

## RESULTS

### Characteristics of the participants according to the exposure doses in the FY2011 CHC

In total, 25,685 residents (10,796 men; 14,889 women) did not receive an EDEE assessment (Table 1). The mean and standard deviation (SD) for each health examination item and the numbers of participants with a health-related disease according to their EDEE assessment status are shown in Table 1. According to a previous report by Ishikawa,^4^ 99.4% of the residents who took part in the movement survey had an EDEE of <3 mSv. Therefore, we classified the subjects into three groups according to EDEE for 4 months after the GEJE: 0 to <1, 1 to <2, and ≥2 mSv and their characteristics are shown according to their EDEE assessment status in Table 2. Among the subjects with complete information on EDEE status, 19,238 (67.7%) had an EDEE of 0 to <1 mSv, 7,089 (25.0%) had an EDEE of 1 to <2 mSv, and 2,075 (7.3%) had an EDEE ≥2 mSv. After imputation of EDEE for the 25,685 residents who did not undergo EDEE assessment, the number of subjects in the same categories as above were 36,525 (67.5%), 13,315 (24.6%) and 4,248 (7.9%), respectively (Table 2). As shown in Table 1 and Table 2, the tendency was essentially the same in both groups with and without EDEE assessment, except for evacuation status.

eTable 1 shows the baseline characteristics of the study subjects classified according to the incident or no incident of each outcome. Subjects with lifestyle-related diseases tended to be older and had the following characteristics: EDEE ≥2 mSv, lived in an evacuation area at the time of the disaster, and receiving medication for hypertension, DM or dyslipidemia.

### Adjusted hazard ratios of lifestyle-related diseases in the CHC from FY2011 to FY2017

The average duration of follow-up during the period from FY2011 to FY2017 ranged from 3.7 to 4.3 years, although it varied slightly by disease. Table 3 shows the adjusted HRs and 95% CIs for the incidence of the various outcome measure for the three exposure doses. After adjustment for age and sex, increasing exposure doses were significantly associated with an increased risk for hypertension, DM, dyslipidemia, hyperuricemia, liver dysfunction, and polycythemia. The age- and sex-adjusted HRs for hypertension, DM, dyslipidemia, hyperuricemia, liver dysfunction, polycythemia, and anemia for the 1 to <2 and ≥2 mSv EDEE groups when compared to the <1 mSv EDEE group are shown in Table 3. After further adjustment for BMI and medication for hypertension, DM, dyslipidemia (only lifestyle-related diseases), smoking habit, heavy alcohol drinking, blood test value(s) at baseline, and evacuation status, the associations disappeared. Basically, incidence of lifestyle-related diseases is affected by age and sex. However, the associations were substantially the same in the present study when stratified by sex or when analyzed by subjects who aged 40–74 years old, except for those with anemia (data not shown).

**Table 3.** Cox regression analyses of various diseases in the comprehensive health checks from FY2012 to FY2017 stratified according to radiation exposure dose

Effective dose	<1 mSv	1 to <2 mSv	≥2 mSv
**Hypertension**			
Number at risk^a^	15,136	5,276	1,710
Number of cases^a^	3,598	1,317	501
Person years^a^	56,796	19,735	5,997
Incidence rate/100,000 pys	6,334.4	6,674.5	8,355.2
Age-, sex-adjusted HRs	1	1.02 (0.95–1.10)	1.29 (1.16–1.44)
Multivariable-adjusted HRs^b^	1	0.97 (0.90–1.04)	1.09 (0.98–1.22)
**Diabetes Mellitus**			
Number at risk^a^	23,391	8,572	2,921
Number of cases^a^	1,585	631	239
Person years^a^	100,257	36,428	12,066
Incidence rate/100,000 pys	1,581.0	1,731.6	1,981.5
Age-, sex-adjusted HRs	1	1.05 (0.93–1.18)	1.17 (1.02–1.36)
Multivariable-adjusted HRs^b^	1	1.04 (0.92–1.18)	1.01 (0.87–1.18)
**Dyslipidemia**			
Number at risk^a^	13,280	4,788	1,627
Number of cases^a^	953	365	144
Person years^a^	53,283	19,042	6,178
Incidence rate/100,000 pys	1,788.4	1,915.8	2,335.6
Age-, sex-adjusted HRs	1	1.06 (0.91–1.22)	1.28 (1.04–1.57)
Multivariable-adjusted HRs^b^	1	1.06 (0.91–1.23)	1.13 (0.91–1.40)
**Kidney disease**			
Number at risk^a^	22,120	8,237	2,848
Number of cases^a^	4,606	1,686	619
Person years^a^	85,122	31,857	10,882
Incidence rate/100,000 pys	5,410.6	5,293.7	5,688.2
Age-, sex-adjusted HRs	1	0.97 (0.91–1.03)	1.04 (0.95–1.13)
Multivariable-adjusted HRs^b^	1	0.96 (0.89–1.02)	1.04 (0.95–1.14)
**Hyperuricemia**			
Number at risk^a^	23,600	8,470	2,846
Number of cases^a^	3,459	1,292	512
Person years^a^	95,609	34,019	11,006
Incidence rate/100,000 pys	3,617.7	3,798.8	4,651.2
Age-, sex-adjusted HRs	1	0.99 (0.92–1.06)	1.16 (1.04–1.29)
Multivariable-adjusted HRs^b^	1	0.98 (0.90–1.06)	1.08 (0.96–1.20)
**Liver dysfunction**			
Number at risk^a^	19,697	6,928	2,130
Number of cases^a^	5,049	1,896	644
Person years^a^	73,869	25,582	7,455
Incidence rate/100,000 pys	6,834.9	7,410.6	8,642.6
Age-, sex-adjusted HRs	1	1.03 (0.97–1.09)	1.17 (1.06–1.29)
Multivariable-adjusted HRs^b^	1	1.02 (0.96–1.09)	1.06 (0.96–1.17)
**Polycythemia**			
Number at risk^a^	22,770	8,281	2,841
Number of cases^a^	487	219	86
Person years^a^	98,319	35,546	11,964
Incidence rate/100,000 pys	495.1	616.1	720.5
Age-, sex-adjusted HRs	1	1.17 (0.99–1.40)	1.32 (1.02–1.71)
Multivariable-adjusted HRs^b^	1	1.08 (0.90–1.30)	1.07 (0.82–1.39)
**Anemia**			
Number at risk^a^	21,748	7,981	2,758
Number of cases^a^	2,957	993	312
Person years^a^	88,915	32,769	11,184
Incidence rate/100,000 pys	3,325.8	3,031.2	2,786.2
Age-, sex-adjusted HRs	1	0.93 (0.86–1.01)	0.88 (0.77–1.01)
Multivariable-adjusted HRs^b^	1	1.04 (0.96–1.14)	1.14 (0.99–1.31)
**Thrombocytopenia**			
Number at risk^a^	22,357	8,186	2,808
Number of cases^a^	916	327	113
Person years^a^	94,972	34,829	11,725
Incidence rate/100,000 pys	964.8	938.9	961.2
Age-, sex-adjusted HRs	1	0.94 (0.81–1.09)	0.95 (0.74–1.23)
Multivariable-adjusted HRs^b^	1	1.00 (0.86–1.15)	1.01 (0.78–1.32)
**Lymphopenia**			
Number at risk^a^	22,902	8,332	2,874
Number of cases^a^	846	301	96
Person years^a^	98,200	35,611	12,098
Incidence rate/100,000 pys	861.7	843.9	796.0
Age-, sex-adjusted HRs	1	0.97 (0.83–1.12)	0.91 (0.73–1.15)
Multivariable-adjusted HRs^b^	1	1.00 (0.86–1.17)	0.95 (0.75–1.20)
**Neutropenia**			
Number at risk^a^	22,637	8,269	2,843
Number of cases^a^	810	273	100
Person years^a^	96,748	35,287	11,888
Incidence rate/100,000 pys	837.4	773.4	840.3
Age-, sex-adjusted HRs	1	0.96 (0.81–1.13)	1.08 (0.86–1.36)
Multivariable-adjusted HRs^b^	1	1.00 (0.84–1.18)	1.21 (0.96–1.53)

pys, person-years; HRs, hazard ratios.

^a^The number at risk, number of cases and person years were averages of the 10 imputed datasets.

^b^Multivariable-adjusted HRs were adjusted for age at baseline, sex, smoking habit, heavy drinking, biochemical value(s) at baseline, and evacuation status. BMI (quartiles) and medication (hypertension, diabetes mellitus, dyslipidemia) were also adjusted for lifestyle-related diseases.

The 95% confidence interval is shown in parentheses.

eTable 2 shows the results of mediation analyses for lifestyle-related diseases after adjustment for BMI or/and evacuation status. BMI and evacuation status contributed substantially and independently to multivariable HRs of lifestyle-related diseases. For example, the HRs of DM for ≥2 mSv EDEE groups compared to the <1 mSv EDEE group were as follows; model 1 (age and sex adjusted): 1.17 (95% CI, 1.02–1.36), model 2a (age, sex and BMI adjusted): 1.06 (95% CI, 0.92–1.23), model 2b (age, sex and evacuation status adjusted): 1.09 (95% CI, 0.94–1.27), model 3 (age, sex, BMI and evacuation status adjusted): 1.00 (95% CI, 0.86–1.16), and model 4 (fully adjusted): 1.01 (95% CI, 0.87–1.18).

Cox regression analyses were also performed only for subjects with data from the basic survey (eTable 3), and the associations were essentially the same; however, the association with anemia was statistically significant. The multivariable-adjusted HRs for the 1 to <2 and ≥2 mSv EDEE groups when compared to the <1 mSv EDEE group were 1.07 (95% CI, 0.98–1.18) and 1.27 (95% CI, 1.08–1.48).

## DISCUSSION

The 2020 report from the United Nations Scientific Committee on the Effects of Atomic Radiation estimated that the maximum EDEE for residents evacuated from the area around the FDNPP for the year after the GEJE was 7.8 mSv for 1-year-olds, 6.5 mSv for 10-year-olds, and 5.5 mSv for adults, and the maximum EDEE for 10 years after the accident in non-evacuated areas of the prefecture was 14 mSv for 1-year-olds, 12 mSv for 10-year-olds, and 11 mSv for adults.^31^ The 2008 report stated that low-dose exposure not exceeding 100 mSv has no confirmed health effects on the human body, including the onset of cancer.^32^

The results of the present analysis revealed a significant relationship between EDEE and the incidence of hypertension, diabetes mellitus, dyslipidemia, hyperuricemia, liver dysfunction, and polycythemia from FY2011 to FY2017 in the age- and sex-adjusted model. However, after further adjustment for evacuation status and lifestyle-related factors, the associations disappeared. A significant relationship was also observed between the incidence of these diseases and evacuation status in the present study (data not shown). We considered that the EDEEs of the residents who lived in areas that were completely evacuated at the time of the GEJE were slightly higher than those of other residents who lived outside the complete evacuation areas, because the EDEE was estimated based on movement surveys for the 4 months after the GEJE.^1^ After the GEJE, residents in the evacuation areas tended to have less physical activity, poor dietary intake of fruits and vegetables, and severe psychological stress.^33^^–^^35^ The association between these factors and the increased risk of metabolic factors, such as abdominal obesity, is the probable reason for the observed association between EDEE and hypertension, diabetes mellitus, dyslipidemia, hyperuricemia, liver dysfunction, and polycythemia via these lifestyle factors. Unfortunately, we did not evaluate lifestyle factors, such as physical activity, diet, and psychological stress, in the present study. On the other hand, previous long-term prospective studies conducted in Hiroshima and Nagasaki atomic bomb survivors have shown that radiation exposure doses were associated with an increased risk of stroke and heart diseases.^36^^,^^37^ However, because the radiation doses of Fukushima residents were much lower than those of the Hiroshima and Nagasaki residents, it is difficult to compare the two subject groups. Yet, 28.9% of the residents in the evacuation area of Fukushima Prefecture still believe that long-term effects of radiation exposure are possible.^38^ Therefore, we also need to examine direct and/or indirect effects of radiation exposure on the risk of stroke and heart diseases in the future.

In the present study, the EDEE was not associated with the incidence of thrombocytopenia, lymphopenia, or neutropenia from FY2011 to FY2017. Among the items evaluated in the CHC as part of the Fukushima Health Management Survey, the WBC count, particularly lymphocyte count, is the most susceptible to radiation exposure, and the relationship between EDEE and decreases in lymphocyte, granulocyte, and platelet counts have already been reported.^39^ The threshold for lymphocyte depletion is thought to be 0.5 Gy (500 mSv), with the number of lymphocytes reportedly decreasing quickly after radiation exposure.^39^ However, the number of lymphocytes does recover following temporary exposure to a EDEE of about 500 mSv, unless the same level of exposure is continued.^40^ Hence, especially in cases with transient low-dose exposure, the effects of radiation exposure immediately after the GEJE cannot be determined from the results of medical examinations performed several months after the GEJE. In fact, no significant relationship between EDEE with neutrophil, lymphocyte, or platelet count or anemia was found in the present study. Radiation exposure can also cause stem cell damage in the bone marrow, which might result in the onset of myelodysplastic syndrome (MDS), characterized by cytopenia, several years later. The relationship between radiation exposure dose and the prevalence of MDS has been previously reported in atomic bomb survivors in Nagasaki.^41^ However, the EDEE of evacuees in Fukushima Prefecture was extremely low when compared to that of the atomic bomb survivors in Nagasaki.

While the EDEE of ≥90% of the residents was <3 mSv in this study, it is doubtful whether it was meaningful to classify subjects into the three EDEE groups for comparisons of the results of the CHC. The highest dose of medical radiation exposure occurs with computed tomography (CT) scans, which is significantly higher than the EDEE in this study. For example, a single CT scan is associated with radiation exposure of 5–60 mSv,^42^ indicating that frequent CT examinations in the medical field might be problematic. However, the health damage caused by a single CT scan is generally not considered an issue. Furthermore, the estimated average individual ionizing radiation dose of ionizing radiation dose per year from all natural radiation sources is 2.4 mSv.^20^ Therefore, the total exposure dose of most residents in the evacuation areas was less than 6 mSv, including the exposure from the natural world, which is not considered to have negative health effects. Meanwhile, in the present study, a significant positive association was found between anemia and radiation dose in multivariate analysis, although no association was found in the sex- and age-adjusted analysis. The FHMS also showed a consistent increase in polycythemia after the earthquake and in the years after the earthquake, which was affected by evacuation and increased overweight people.^16^ In the present multivariable analysis, evacuation and BMI were added as adjustment variables, which might have resulted in over-adjustment of anemia.

Several limitations must be considered when interpreting the results of this study. First, in the CHC, the participation rate of residents was relatively low (30%), so the results of this study might not be representative of the entire population. In addition, only about half of the residents who participated in the CHC had data on EDEE. However, the results of the present study, supplemented using the MI method, were very similar to the results of the analysis using only those who had data, indicating that the effect of the participation rate on the results of the analysis is considered to be small. Second, since the CHC was started several months after the GEJE, data immediately after the GEJE are not available. Therefore, the data used in the present study did not reflect the effects of the acute phase in terms of radiation exposure and might have been affected by earthquake-related factors, such as an evacuation. Third, the maximum follow-up period for this study was only 6 years. Longer follow-up may be required to assess the effects of radiation on development of diseases. Finally, as also mentioned above, we did not examine the effects of lifestyle factors, such as diet and physical activity, and psychological stress on the associations between EDEE and lifestyle-related diseases in the present study.

In conclusion, we analyzed the relationship between EDEE and health effects in the residents of evacuated areas of Fukushima Prefecture and found that EDEE was not directly associated with the incidence of lifestyle-related diseases. As reported by the United Nations Scientific Committee on the Effects of Atomic Radiation in 2008, the EDEE due to the accident at the FDNPP was insufficient to cause problematic health effects, and previous studies have also reported that residents who were exposed to higher radiation levels after the accident tended to change their lifestyles after evacuation. Therefore, we speculate that the residents with higher external radiation doses in Fukushima Prefecture might suffer from lifestyle-related diseases due to evacuation and the subsequent lifestyle changes, rather than the direct effects of radiation exposure.

## SUPPLEMENTARY MATERIAL

eTable 1. Baseline characteristics and covariates in Cox proportional hazards model analyses according to incident or no incident of each outcome

eTable 2. Hazard ratios (HRs) and 95% confidence intervals for lifestyle-related diseases in the comprehensive health checks from FY2012 to FY2017 according to radiation exposure

eTable 3. Cox proportional hazards model analysis of diseases in the comprehensive health checks from FY2012 to FY2017 (complete case analyses) stratified according to radiation exposure dose
