# Supplementary material for: Effects of External Radiation Exposure Resulting From the Fukushima Daiichi Nuclear Power Plant Accident on the Health of Residents in the Evacuation Zones: the Fukushima Health Management Survey
Source: J Epidemiol. 2022 Dec 5;32(Suppl 12):S84–94. doi: 10.2188/jea.JE20210286 (PMC9703929; doi:10.2188/jea.JE20210286)
Supplement: Supplementary file 1 [file je-32-S084-s001.pdf]

eTable 1. Baseline characteristics and covariates in Cox proportional hazards model analyses according to incident or no incident of each outcome

	Hypertension		Diabetes mellitus		Dyslipidemia		Kidney disease		Hyperuricemia		Liver dysfunction	
	No incident	Incident	No incident	Incident	No incident	Incident	No incident	Incident	No incident	Incident	No incident	Incident
Subjects, n (%)	16,706 (75.5)	5,416 (24.5)	32,430 (93.0)	2,455 (7.0)	18,233 (92.6)	1,462 (7.4)	26,294 (79.2)	6,911 (20.8)	29,654 (84.9)	5,263 (15.1)	21,167 (73.6)	7,589 (26.4)
EDEE, n (%)												
: <1 mSv	11,538 (69.1)	3,598 (66.4)	21,806 (67.2)	1,585 (64.6)	12,327 (67.6)	953 (65.2)	17,514 (66.6)	4,606 (66.6)	20,142 (67.9)	3,459 (65.7)	14,649 (69.2)	5,049 (66.5)
: 1–2 mSv	3,959 (23.7)	1,317 (24.3)	7,941 (24.5)	631 (25.7)	4,423 (24.3)	365 (25.0)	6,551 (24.9)	1,686 (24.4)	7,178 (24.2)	1,292 (24.6)	5,033 (23.8)	1,896 (25.0)
: ≥2 mSv	1,209 (7.2)	501 (9.3)	2,682 (8.3)	239 (9.7)	1,482 (8.1)	144 (9.9)	2,229 (8.5)	619 (9.0)	2,334 (7.9)	512 (9.7)	1,486 (7.0)	644 (8.5)
Men, n (%)	5,325 (31.9)	2,328 (43.0)	12,090 (37.3)	1,302 (53.0)	7,324 (40.2)	692 (47.3)	10,383 (39.5)	2,807 (40.6)	9,784 (33.0)	2,819 (53.6)	5,546 (26.2)	3,397 (44.8)
Age at baseline (years), mean (SD)	44.6 (16.4)	59.2 (12.9)	53.9 (17.2)	63.1 (11.8)	50.4 (18.4)	64.9 (10.5)	50.9 (16.7)	63.2 (12.4)	54.3 (17.2)	58.9 (15.3)	53.4 (18.3)	57.5 (15.6)
BMI (kg/m^2), mean (SD)	22.3 (3.4)	23.9 (3.4)	23.3 (3.5)	25.5 (3.6)	22.6 (3.5)	24.5 (3.5)	23.3 (3.7)	24.1 (3.5)	23.1 (3.5)	24.8 (3.6)	22.7 (3.3)	23.7 (3.5)
Smoking habitus, n (%)	3,195 (19.1)	952 (17.6)	5,267 (16.2)	399 (16.3)	3,218 (17.6)	191 (13.1)	4,918 (18.7)	846 (12.2)	4,385 (14.8)	1,053 (20.0)	2,726 (12.9)	1,216 (16.0)
Heavy drinking, n (%)	455 (2.7)	275 (5.1)	1,495 (4.6)	165 (6.7)	1,100 (6.0)	112 (7.7)	1,405 (5.3)	335 (4.8)	961 (3.2)	408 (7.8)	330 (1.6)	327 (4.3)
Living in the following areas at the time of disaster												
Evacuation areas,[a] n (%)	8,628 (51.6)	3,102 (57.3)	17,061 (52.6)	1,483 (60.4)	9,465 (51.9)	891 (60.9)	13,766 (52.4)	3,945 (57.1)	15,538 (52.4)	3,044 (57.8)	10,512 (49.7)	4,409 (58.1)
Evacuation order lifted by the end of 2016,[b] n (%)	10,009 (59.9)	2,992 (55.2)	19,232 (59.3)	1,344 (54.7)	10,809 (59.3)	812 (55.5)	15,585 (59.3)	3,897 (56.4)	17,624 (59.4)	2,905 (55.2)	12,960 (61.2)	4,249 (56.0)
Medication												
: Hypertension, n (%)	0 (0.0)	0 (0.0)	8,164 (25.2)	1,210 (49.3)	3,865 (21.2)	783 (53.6)	5,518 (21.0)	2,898 (41.9)	7,231 (24.4)	2,220 (42.2)	5,140 (24.3)	2,398 (31.6)
: Diabetes mellitus, n (%)	324 (1.9)	261 (4.8)	0 (0.0)	0 (0.0)	558 (3.1)	185 (12.7)	1,036 (3.9)	508 (7.4)	1,443 (4.9)	392 (7.4)	926 (4.4)	428 (5.6)
: Dyslipidemia, n (%)	1,015 (6.1)	642 (11.9)	4,104 (12.7)	621 (25.3)	0 (0.0)	0 (0.0)	2,946 (11.2)	1,495 (21.6)	4,070 (13.7)	993 (18.9)	2,656 (12.5)	1,260 (16.6)
Laboratory data												
: variable 1	115.3 (11.8)	126.2 (8.9)	5.2 (0.3)	5.8 (0.4)	64.3 (14.8)	59.0 (13.9)	83.4 (13.8)	69.5 (10.0)	4.5 (1.0)	5.7 (0.8)	19.5 (4.0)	21.8 (4.3)
: variable 2	70.4 (9.1)	77.1 (7.4)			106.0 (20.6)	113.7 (20.9)					14.9 (5.1)	18.6 (5.5)
: variable 3											18.4 (8.0)	25.6 (10.8)

eTable 1 (continued).

	Polycythemia		Anemia		Thrombocytopenia		Lymphocytopenia		Neutropenia	
	No incident	Incident	No incident	Incident	No incident	Incident	No incident	Incident	No incident	Incident
Subjects, n (%)	32,718 (97.7)	780 (2.3)	28,151 (87.2)	4,119 (12.8)	31,663 (96.0)	1,312 (4.0)	32,567 (96.6)	1,155 (3.4)	32,231 (96.6)	1,134 (3.4)
EDEE, n (%)										
: <1 mSv	22,022 (67.3)	479 (61.4)	18,737 (66.6)	2,862 (69.5)	21,216 (67.0)	887 (67.6)	21,851 (67.1)	785 (68.0)	21,602 (67.0)	774 (68.2)
: 1–2 mSv	7,975 (24.4)	215 (27.6)	6,971 (24.8)	959 (23.3)	7,779 (24.6)	318 (24.2)	7,960 (24.4)	284 (24.6)	7,914 (24.6)	264 (23.3)
: ≥2 mSv	2,722 (8.3)	85 (10.9)	2,443 (8.7)	297 (7.2)	2,669 (8.4)	107 (8.1)	2,756 (8.5)	86 (7.4)	2,715 (8.4)	96 (8.5)
Men, n (%)	13,602 (41.6)	462 (59.2)	12,637 (44.9)	1,379 (33.5)	13,102 (41.4)	685 (52.2)	13,788 (42.3)	523 (45.3)	13,950 (43.3)	310 (27.3)
Age at baseline (years), mean (SD)	55.8 (16.8)	53.2 (16.1)	54.9 (16.7)	61.3 (16.1)	55.0 (16.9)	65.8 (12.4)	55.6 (16.8)	59.5 (15.9)	55.7 (16.9)	55.7 (15.7)
Smoking habitus, n (%)	5,309 (16.2)	209 (26.8)	5,178 (18.4)	371 (9.0)	5,397 (17.0)	150 (11.4)	5,581 (17.1)	107 (9.3)	5,574 (17.3)	78 (6.9)
Heavy drinking, n (%)	1,645 (5.0)	68 (8.7)	1,580 (5.6)	151 (3.7)	1,612 (5.1)	84 (6.4)	1,710 (5.3)	59 (5.1)	1,685 (5.2)	60 (5.3)
Living in the following areas at the time of disaster										
Evacuation areas,[a] n (%)	17,522 (53.6)	500 (64.1)	15,266 (54.2)	2,042 (49.6)	16,924 (53.5)	732 (55.8)	17,499 (53.7)	643 (55.7)	17,381 (53.9)	595 (52.5)
Evacuation order lifted by the end of 2016,[b] n (%)	19,260 (58.9)	373 (47.8)	16,297 (57.9)	2,589 (62.9)	18,488 (58.4)	759 (57.9)	19,090 (58.6)	677 (58.6)	18,812 (58.4)	695 (61.3)
Laboratory data										
: variable 1	466.6 (40.3)	523.4 (34.8)	14.7 (1.3)	13.4 (1.0)	252.1 (54.3)	182.5 (32.1)	2179.8 (624.9)	444.6 (350.1)	3344.2 (1210.2)	305.1 (862.2)
: variable 2	14.3 (1.4)	15.8 (1.3)								
: variable 3	42.7 (3.6)	46.9 (3.1)								

[a] All area of Hirono, Naraha, Tomioka, Kawauchi, Okuma, Futaba, Namie, Katsurao, and Iitate, and part of Tamura, Minami-Soma, Kawamata, and Date.

[b] Hirono, Date, Tamura, Naraha, Kawauchi, Katsurao and Minami-Soma.

Variable1:
SBP (hypertension),
HbA1c (diabetes mellitus),
HDL (dyslipidemia),
eGFR (kidney disease),
uric acid (hyperuricemia),
AST (liver dysfunction),
RBC (polycythemia),
Hb (anemia),
PLT (hrombocytopenia),
lymphocyte count (lymphopenia)
or neutrophil count (neutropenia).

Variable2:
DBP (hypertension),
LDL (dyslipidemia),
ALT (liver dysfunction)
or Hb (polycythemia).

Variable3:
γ-GTP (liver dysfunction)
or Ht (polycythemia).

eTable 2. Hazard ratios (HRs) and 95% confidence intervals for lifestyle-related diseases in the comprehensive health checks from FY2012 to FY2017 according to radiation exposure

Effective dose	<1mSv	1-2mSv	≥2mSv
**Hypertension**			
Model 1 HRs	1	1.02(0.95-1.10)	1.29(1.16-1.44)
Model 2a HRs	1	1.01(0.94-1.09)	1.20(1.08-1.33)
Model 2b HRs	1	1.01(0.94-1.08)	1.22(1.09-1.36)
Model 3 HRs	1	0.99(0.92-1.07)	1.13(1.01-1.26)
Model 4 HRs	1	0.97(0.90-1.04)	1.09(0.98-1.22)
**Diabetes Mellitus**			
Model 1 HRs	1	1.05(0.93-1.18)	1.17(1.02-1.36)
Model 2a HRs	1	1.03(0.91-1.16)	1.06(0.92-1.23)
Model 2b HRs	1	1.04(0.92-1.17)	1.09(0.94-1.27)
Model 3 HRs	1	1.02(0.90-1.15)	1.00(0.86-1.16)
Model 4 HRs	1	1.04(0.92-1.18)	1.01(0.87-1.18)
**Dyslipidemia**			
Model 1 HRs	1	1.06(0.91-1.22)	1.28(1.04-1.57)
Model 2a HRs	1	1.06(0.91-1.22)	1.20(0.98-1.46)
Model 2b HRs	1	1.05(0.91-1.22)	1.19(0.95-1.47)
Model 3 HRs	1	1.05(0.91-1.22)	1.12(0.90-1.39)
Model 4 HRs	1	1.06(0.91-1.23)	1.13(0.91-1.40)
**Kidney disease**			
Model 1 HRs	1	0.97(0.91-1.03)	1.04(0.95-1.13)
Model 2a HRs	1	0.96 (0.91–1.02)	1.01 (0.92–1.10)
Model 2b HRs	1	0.96 (0.90–1.02)	0.99 (0.90–1.08)
Model 3 HRs	1	0.95 (0.90–1.01)	0.96 (0.88–1.06)
Model 4 HRs	1	0.96 (0.89–1.02)	1.04 (0.95–1.14)
**Hyperuricemia**			
Model 1 HRs	1	0.99 (0.92–1.06)	1.16 (1.04–1.29)
Model 2a HRs	1	0.98 (0.91–1.05)	1.09 (0.98–1.22)
Model 2b HRs	1	0.98 (0.91–1.05)	1.11 (0.99–1.24)
Model 3 HRs	1	0.97 (0.90–1.04)	1.05 (0.94–1.18)
Model 4 HRs	1	0.98 (0.90–1.06)	1.08 (0.96–1.20)
**Liver dysfunction**			
Model 1 HRs	1	1.03 (0.97–1.09)	1.17 (1.06–1.29)
Model 2a HRs	1	1.03 (0.97–1.09)	1.13 (1.03–1.24)
Model 2b HRs	1	1.02 (0.96–1.09)	1.08 (0.98–1.19)
Model 3 HRs	1	1.02 (0.96–1.08)	1.05 (0.95–1.15)
Model 4 HRs	1	1.02 (0.96–1.09)	1.06 (0.96–1.17)

HRs, hazard ratios.

95% confidence interval is shown in parentheses.

[a] Model 1: Adjusted for age at baseline and sex.

[b] Model 2a: Adjusted for model 1 + BMI (quartiles).

[c] Model 2b: Adjusted for model 1 + evacuation status.

[d] Model 3: Adjusted for model 1 + BMI (quartiles) and evacuation status.

[e] Model 4: Fully adjusted model, as in table 3.

eTable 3. Cox proportional hazards model analysis of diseases in the comprehensive health checks from FY2012 to FY2017 (complete case analyses) stratified according to radiation exposure dose

Effective dose	<1 mSv	1–2 mSv	≥2 mSv
**Hypertension**			
Number at risk	8,616	3,026	847
Number of cases	2,074	776	266
Person years	32,741	11,503	3,047
Incidence rate /100,000 pys	6,334.6	6,746.3	8,728.5
Age-, sex-adjusted HRs	1	1.03 (0.95–1.12)	1.33 (1.17–1.51)
Multivariable-adjusted HRs[a]	1	0.96 (0.88–1.04)	1.04 (0.91–1.19)
**Diabetes mellitus**			
Number at risk	13,203	4,964	1,510
Number of cases	914	378	131
Person years	57,683	21,555	6,425
Incidence rate /100,000 pys	1,584.5	1,753.7	2,038.8
Age-, sex-adjusted HRs	1	1.06 (0.94–1.20)	1.20 (1.00–1.44)
Multivariable-adjusted HRs[a]	1	1.04 (0.92–1.18)	1.02 (0.84–1.23)
**Dyslipidemia**			
Number at risk	7,467	2,754	851
Number of cases	584	222	81
Person years	30,357	11,120	3,326
Incidence rate /100,000 pys	1,923.8	1,996.5	2,435.0
Age-, sex-adjusted HRs	1	1.03 (0.88–1.20)	1.21 (0.96–1.53)
Multivariable-adjusted HRs[a]	1	1.03 (0.88–1.20)	1.05 (0.82–1.34)
**Kidney disease**			
Number at risk	12,507	4,763	1,478
Number of cases	2,701	1,007	350
Person years	48,526	18,683	5,824
Incidence rate /100,000 pys	5,566.1	5,390.1	6,009.3
Age-, sex-adjusted HRs	1	0.95 (0.89–1.03)	1.04 (0.93–1.16)
Multivariable-adjusted HRs[a]	1	0.93 (0.86–1.00)	1.05 (0.93–1.18)
**Hyperuricemia**			
Number at risk	13,361	4,893	1,469
Number of cases	1,991	745	284
Person years	55,021	20,027	5,822
Incidence rate /100,000 pys	3,618.6	3,719.9	4,878.1
Age-, sex-adjusted HRs	1	0.98 (0.90–1.07)	1.22 (1.07–1.38)
Multivariable-adjusted HRs[a]	1	0.98 (0.90–1.07)	1.07 (0.94–1.22)
**Liver dysfunction**			
Number at risk	11,159	4,030	1,078
Number of cases	2,972	1,128	331
Person years	42,327	15,063	3,870
Incidence rate /100,000 pys	7,021.6	7,488.7	8,552.2
Age-, sex-adjusted HRs	1	1.03 (0.96–1.11)	1.13 (1.01–1.26)
Multivariable-adjusted HRs[a]	1	1.02 (0.95–1.10)	0.98 (0.87–1.10)

eTable 3 (continued).

Effective dose	<1 mSv	1–2 mSv	≥2 mSv
**Polycythemia**			
Number at risk	12,855	4,760	1,476
Number of cases	279	132	41
Person years	56,529	20,924	6,422
Incidence rate /100,000 pys	493.6	630.9	638.5
Age-, sex-adjusted HRs	1	1.22 (1.00–1.51)	1.18 (0.85–1.65)
Multivariable-adjusted HRs[a]	1	1.14 (0.92–1.40)	1.00 (0.71–1.40)
**Anemia**			
Number at risk	12,276	4,552	1,438
Number of cases	1,700	597	177
Person years	51,106	19,110	5,980
Incidence rate /100,000 pys	3,326.4	3,124.0	2,959.7
Age-, sex-adjusted HRs	1	0.95 (0.86–1.04)	0.94 (0.80–1.09)
Multivariable-adjusted HRs[a]	1	1.07 (0.98–1.18)	1.27 (1.08–1.48)
**Thrombocytopenia**			
Number at risk	12,568	4,685	1,467
Number of cases	546	194	75
Person years	54,285	20,419	6,290
Incidence rate /100,000 pys	1,005.8	950.1	1,192.3
Age-, sex-adjusted HRs	1	0.92 (0.78–1.08)	1.12 (0.88–1.42)
Multivariable-adjusted HRs[a]	1	0.99 (0.84–1.17)	1.18 (0.92–1.51)
**Lymphopenia**			
Number at risk	12,922	4,770	1,492
Number of cases	543	199	57
Person years	56,343	20,835	6,479
Incidence rate /100,000 pys	963.7	955.1	879.8
Age-, sex-adjusted HRs	1	0.98 (0.83–1.15)	0.90 (0.68–1.18)
Multivariable-adjusted HRs[a]	1	1.02 (0.86–1.20)	0.93 (0.71–1.23)
**Neutropenia**			
Number at risk	12,766	4,724	1,476
Number of cases	484	168	54
Person years	55,510	20,616	6,381
Incidence rate /100,000 pys	871.9	814.9	846.3
Age-, sex-adjusted HRs	1	0.96 (0.81–1.14)	1.05 (0.79–1.39)
Multivariable-adjusted HRs[a]	1	0.98 (0.82–1.17)	1.18 (0.88–1.57)

HRs, hazard ratios; pys, person-years.

[a] Multivariable-adjusted HRs were adjusted for age at baseline, sex, smoking habit, heavy drinking, biochemical value(s) at baseline and evacuation status. BMI (quartiles) and medication (hypertension, diabetes mellitus, dyslipidemia) were also adjusted for lifestyle-related diseases.

The 95% confidence interval is in parentheses.
